# Mediterranean Style Dietary Pattern with High Intensity Interval Training in Men with Prostate Cancer Treated with Androgen Deprivation Therapy: A Pilot Randomised Control Trial

**DOI:** 10.3390/ijerph19095709

**Published:** 2022-05-07

**Authors:** Brenton J. Baguley, Kirsten Adlard, David Jenkins, Olivia R. L. Wright, Tina L. Skinner

**Affiliations:** 1 Institute for Physical Activity and Nutrition, Deakin University, 221 Burwood Highway, Burwood, VIC 3125, Australia; 2 School of Human Movement and Nutrition Sciences, The University of Queensland, Brisbane, QLD 4067, Australia; k.adlard@uq.edu.au (K.A.); djenkins@usc.edu.au (D.J.); o.wright@uq.edu.au (O.R.L.W.); t.skinner@uq.edu.au (T.L.S.); 3 School of Health and Behavioural Sciences, University of the Sunshine Coast, Maroochydore, QLD 4558, Australia; 4 Applied Sports Science Technology and Medicine Research Centre, Swansea University, Swansea SA1 8EN, Wales, UK; 5 Mater Research Institute, The University of Queensland, Brisbane, QLD 4101, Australia

**Keywords:** mediterranean diet, high intensity interval training, prostate cancer, androgen deprivation therapy

## Abstract

**Background:** Androgen deprivation therapy (ADT) in prostate cancer has been shown to deteriorate body composition (reduced lean mass and increased body and fat mass) and increase the risk of cardiovascular morbidity. The Mediterranean style dietary pattern (MED-diet) and high intensity interval training (HIIT) may synergistically alleviate these side effects and improve quality of life in men treated with ADT. **Methods:** Twenty-three men (65.9 ± 7.8 years; body mass index: 29.6 ± 2.7 kg/m^2^; ADT duration: 33.8 ± 35.6 months) receiving ADT for ≥3 months were randomly assigned (1:1) to 20 weeks of usual care or the MED-diet (10 nutrition consults) with HIIT (4 × 4 min 85–95% heart rate peak, 3× week, starting at 12 weeks). **Results:** The MED-diet with HIIT significantly improved cardiorespiratory fitness (+4.9 mL·kg^−1^·min, *p* < 0.001), and body mass (−3.3 kg, *p* < 0.001) compared to the usual care group at 20 weeks. Clinically meaningful (≥3 points) improvements were seen in quality of life and cancer-related fatigue after 20 weeks. **Conclusions:** The MED-diet with HIIT increased cardiorespiratory fitness and reduced body weight in men with prostate cancer treated with ADT. Larger trials determining whether the MED-diet with HIIT translates to cardiovascular benefits are warranted.

## 1. Introduction

Prostate cancer is the most diagnosed cancer in Australian men (excluding non-melanoma skin cancer) and by 2040 it is predicted that the number of men diagnosed, treated and living with prostate cancer will triple (~370,000) [1]. The use of androgen deprivation therapy (ADT) has coincided with improvements in disease control and improved overall survivorship in many men treated for prostate cancer [2]. ADT is however accompanied with persistent side effects, including an increase in body weight, fat mass and a reduction in lean muscle mass [3,4]. These changes have been associated with an increased risk of coronary heart disease, myocardial infarction and cardiac mortality [5]. Exercise and nutrition interventions during and/or after ADT are strongly recommended to mitigate or improve body composition and reduce cardiometabolic side effects [6,7,8,9]. Herein, lifestyle interventions (i.e., nutrition and exercise) that aim to improve cardiovascular health and body composition are clinically important for long-term health and quality of life in men treated with ADT.

The health benefits from aerobic high intensity interval training (HIIT) in adults with cancer have been extensively reviewed and shown to significantly improve cardiorespiratory fitness ([$\overset{\cdot }{V}$O_2peak_]; MD 2.11 mL·kg^−1^·min^−1^, 95% CI 0.75–3.47, *p* = 0.002) compared to usual care when implemented prior to or after cancer treatment [10]. Compared to moderate-intensity continuous aerobic exercise (150 mins 50–70% maximum heart rate [HR_max_]) HIIT offers similar adaptations in cardiorespiratory fitness in adults with cancer within a shorter time commitment [11]. In adults with colorectal cancer, 4 weeks of HIIT comprising of 4 × 4 min at 85–95% HR_peak_, has been shown to elicit greater improvements in cardiorespiratory fitness compared to moderate-intensity continuous exercise training (+3.5 vs. 0.9 mL·kg^−1^·min^−1^; *p* = 0.016) [12]. These findings further indicate the physiological adaptations from HIIT appear central to the dose of exercise ≥80% HR_max_ [13]. Whilst the evidence showing benefits of HIIT is growing for adults with cancer, its efficacy in men with prostate cancer treated with ADT, where cardiovascular health is of clinical importance, is yet to be investigated.

Meta-analysis of adherence to a Mediterranean diet (MED-diet) in cohort studies has determined that a 2-point increase in adherence score (range: 0–18, higher scores indicating higher adherence) is associated with an 8% reduction in total mortality (RR = 0.92, 95% CI; 0.91–0.93) and a 10% reduction in cardiovascular disease (RR = 0.90, 95% CI; 0.87–0.92) [14]. Furthermore, the MED-diet is associated with a 22% reduction in all-cause mortality in men with prostate cancer [15]. The diverse range of phytochemicals with antioxidant and anti-inflammatory properties within the MED-diet may at least in part contribute to cardiovascular and survivorship benefits for men with prostate cancer [16]. In women with breast cancer, the MED-diet compared to a usual care group following the World Cancer Research Fund healthy eating recommendations significantly reduced body weight (−2.4 vs. −0.9 kg), triglycerides (−14.0 vs. −8.2 mg/dL) and fasting glucose (−1.7 vs. −0.5 mg/dL) [17]. We have previously demonstrated that the MED-diet is safe, feasible and efficacious in reducing cancer-related fatigue, body weight and fat mass in men with prostate cancer treated with ADT [18], with similar findings seen in women with breast cancer [19]. Given the benefits of the MED-diet on body composition and cardiovascular health [18,19,20], and the substantial improvement in cardiorespiratory fitness from HIIT (typically 8-weeks duration) [10], the synergistic effect of the MED-diet and HIIT may offer targeted strategies to mitigate a number of well documented side-effects from ADT. Herein, this study aimed to examine the combined effects of a MED-diet and HIIT on cardiorespiratory fitness, body composition and quality of life, compared to usual care, in men with prostate cancer treated with ADT.

## 2. Materials and Methods

### 2.1. Study Design and Randomisation

A two-arm randomised controlled trial was conducted in accordance with the CONSORT guidance for pilot clinical research study designs [21]. The protocol for this study has been reported elsewhere [22]. Eligibility included: (a) aged ≥18 years, (b) non-smoker, or have quit smoking for ≥3 months, (c) a diagnosis of prostate cancer and treated with ADT for ≥3 months, (d) BMI 18.5–34.9 kg/m^2^.

**Figure 1:**
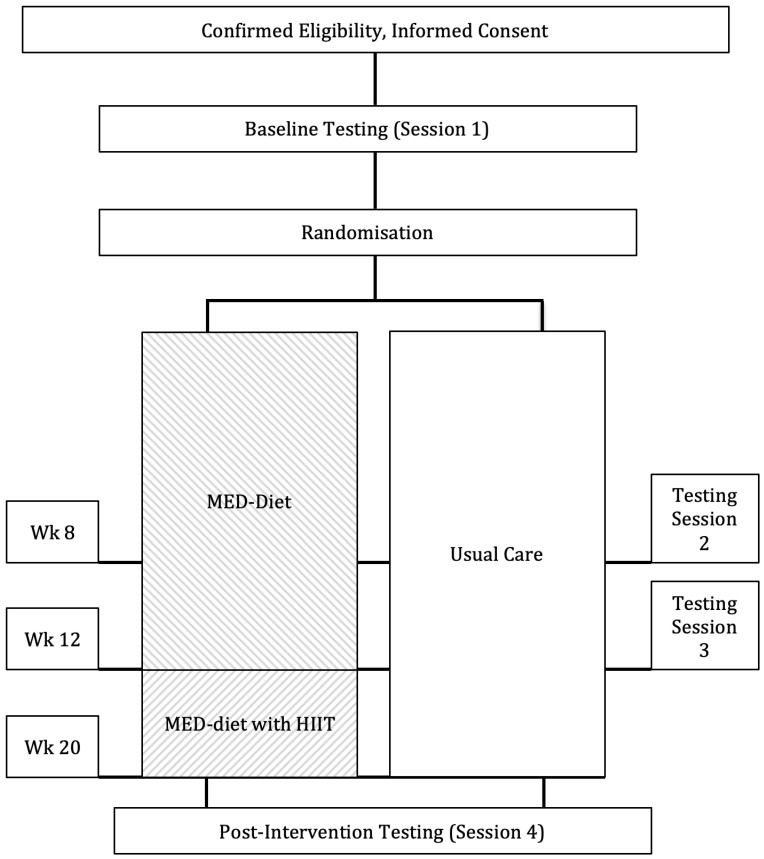
CONSORT diagram and schematic representation of the study. Legend: MED-diet = Mediterranean-style dietary pattern; HIIT = high intensity interval training; Wk = week.

After baseline testing, participants were randomly allocated to either an intervention or usual care group in a 1:1 ratio (Figure 1). Randomisation was completed by a person external to the study using a computerised random number generator with equal probability (Sealedenvelope Ltd., London, UK 2012). Participant concealment was revealed to the investigators only after baseline testing. Figure 1 shows that participants allocated to the intervention received the MED-diet for the entire 20 weeks, and at 12 weeks were asked to complete a HIIT program for 8 weeks. The results from baseline to 12 weeks for the MED-diet and usual care groups have been published elsewhere [18]. Ethical approval was granted by the Human Research Ethics Committee of The University of Queensland (2015001245), The Mater Health Services Human Research Ethics Committee (HREC/15/MHS/38) and was registered with the Australian and New Zealand Clinical Trials Registry (ACTRN12615000512527).

### 2.2. Intervention Nutrition and Exercise Prescription

Participants randomised to the intervention group completed face-to-face, 30 to 45-min nutrition consultations with an Accredited Practising Dietitian every two weeks for 20 weeks. Participants were prescribed an individualised MED-diet outlined previously [22]. From weeks 12–20, participants allocated to the intervention group visited The University of Queensland’s School of Human Movement and Nutrition Sciences exercise laboratory three times per week to complete HIIT sessions. Prior to each exercise training session, each participant’s heart rate and blood pressure were measured and assessed for contraindications to commence exercising as outlined by the American College of Sports Medicine [23]. Exercise intensity was set at 85–95% HR_peak_ and each 4-min interval was interspersed with 3-min of active recovery at 50–70% HR_peak_. Heart rate zones of 50–70% and 85–95% were individually determined from the highest HR recorded during a $\overset{\cdot }{V}$O_2peak_ test at 12 weeks. The HIIT sessions commenced with 10 min of warm up at 50–70% HR_peak_ before participants completed 4 × 4-min bouts of cycling on an air- and magnetically braked cycle ergometer (Wattbike Ltd., Nottingham, UK).

### 2.3. Usual Care (Control Group)

Participants randomised to the usual care group continued their usual medical care during this period. Participants in the usual care group were monitored for 20 weeks and completed testing for the primary and secondary outcome measures at the same time points as outlined below for the intervention group.

### 2.4. Outcomes

#### Cardiorespiratory Fitness

Cardiorespiratory fitness was assessed using a $\overset{\cdot }{V}$O_2peak_ test; the test involved a modified ramp protocol described by Wasserman et al. [24] on a cycle ergometer. Participants began with 3 min of rest for respiratory normalisation, followed by 4 min of warm-up at a resistance of 50 Watts. The electrical resistance provided by the cycle ergometer then increased incrementally by 20–30 W·min^−1^ and participants cycled at a cadence between 60–70 revolutions per minute throughout the test. Heart rate was continuously recorded throughout the exercise using a heart rate monitor (Polar FT1; Polar, Kempele, Finland) and blood pressure (Durashock Sphygmomanometer; Welch Allyn, NY, USA) was recorded every 2 min throughout the test. At the conclusion of each minute, participants indicated their rating of perceived exertion (RPE) on the Borg 6–20 scale [25]. The test was terminated when the participant reached volitional fatigue or at the discretion of the researchers with consideration for exercise testing termination criteria as outlined by the American Association of Cardiovascular and Pulmonary Rehabilitation [26]. The gas analysers and ventilometer were calibrated prior to and verified after each test. Sampled expired air was measured every 15 s using a turbine ventilometer (Morgan, Model 096, Kent, UK). $\overset{\cdot }{V}$O_2peak_ was recorded as the highest $\overset{\cdot }{V}$O_2_ reading averaged over two consecutive readings.

### 2.5. Physical Traits and Body Composition

Height and body mass were measured according to the International Society for the Advancement of Kinanthropometry procedures [27]. Body composition (fat mass, lean mass and body fat mass) was assessed using dual energy X-ray absorptiometry (Hologic Discovery A, Waltham, MA, USA).

### 2.6. Intervention Fidelity

Intervention fidelity was defined by four components: (a) intervention safety, (b) study completion rate, (c) consult attendance rate and (d) adherence to the MED-diet.

(a) All intervention and usual care adverse events were reported to the primary investigator as a measure of intervention safety. Adverse events were defined as an untoward injury or medical occurrence from the intervention (MED-diet) or outcome measures which interfered with the capacity of the participant to carry out their usual activities. Adverse events were also monitored at each nutrition consultation for common signs and symptoms of dietary change (e.g., gastrointestinal tolerance).
(b) Intervention completion was measured by the number of participants completing baseline, 12- and 20-week testing sessions.
(c) Attendance at the MED-diet consultations was measured by the number of sessions attended, divided by the number of sessions prescribed.
(d) Adherence to the MED-diet was measured by the Mediterranean-diet adherence screener (MEDAS) [28]; a 14-question (yes/no) response to the frequency of food groups was included in the MED-diet. The total responses of ‘yes’ were tallied to provide an overall dietary adherence to the MED-diet. Question 8 (how much wine do you drink; ≥7 glasses = adherence) was omitted from the MEDAS due to the intervention promoting a reduction in alcohol intake. High adherence was classified as meeting a priori cut point of ≥75% of the 13-question MEDAS.
(e) Adherence to exercise intensity was quantified by two measures. Mean HR was calculated using a combination of data points collected throughout the exercise intervals and recovery periods. Mean HR was calculated using all data points collected at 1-s epochs throughout the designated interval or recovery period. Time to reach 85% of HR_peak_ was measured at the start of each interval and recorded in minutes: seconds. Adherence was determined by reaching 85% HR_peak_ and quantified by the time spent exercising ≥85% HR_peak_ for each interval. Rating of perceived exertion (RPE) for each interval was also used as a measure of exercise intensity.

### 2.7. Cancer-Related Fatigue and Quality of Life

Cancer-related fatigue and quality of life were measured using the Functional Assessment of Chronic Illness Therapy: Fatigue (FACIT-F), FACIT-general (FACIT-G) questionnaire [29], and the Medical Outcomes Study 36-Item Short-Form Health Survey (SF−36) [30]. The questionnaire instructions were read to the participant by a study investigator (B.J.B), and participants completed the questionnaires in a separate room to the study investigators.

### 2.8. Change in Dietary Intake

Participants completed the Wollongong Dietary Inventory [31], a comprehensive dietary history of intake over the previous month, with cross-checking quantification from the dietitian.

### 2.9. Statistical Analysis

All analyses were conducted in SPSS (version 23.0; Chicago, IL, USA). Intention to treat linear mixed models were used to determine changes in cardiorespiratory fitness, body composition and quality of life between the MED-diet and usual care groups at baseline, 12 weeks and 20 weeks. Models included group, time and group x time as fixed factors, and a random intercept term for each participant in the study to account for the correlation between repeated observations on an individual. All models also adjusted for baseline values by inclusion as a covariate. Model residuals were assessed for normality using the Shapiro–Wilk test and visual inspection of the histogram and quantile-quantile plots. Statistical significance was two-tailed and accepted at the *p* ≤ 0.05 level. For FACIT-F and FACIT-G questionnaires, a ≥3 point change in mean score was classified as a clinically important change [32].

## 3. Results

**Figure 2:**
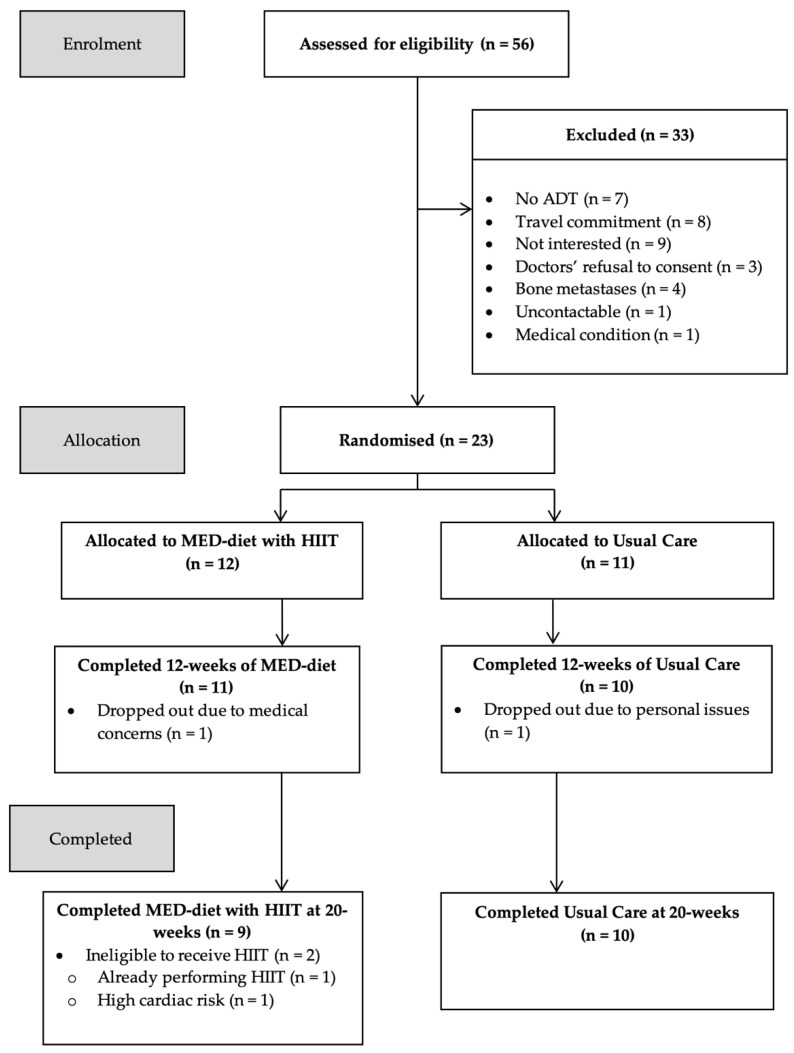
CONSORT diagram illustrating participant flow through the intervention. ADT: androgen deprivation therapy; MED-diet: Mediterranean-style dietary pattern; HIIT: high intensity interval training.

A total of 23 men with prostate cancer treated with ADT were randomised for this trial. Participant flow through the intervention is shown in Figure 1 and the participant characteristics are described in Table 1. As described elsewhere [18], time since diagnosis was longer in the MED-diet with HIIT group (77.1 ± 58.8 months) compared to the usual care group (51.3 ± 42.4 months); however, time on ADT was similar between groups (MED- diet: 36.4 ± 38.3 months; usual care: 31.0 ± 32.2 months). The mean Gleason score for all men in this trial was 8.4 and represents an ISUP (International Society of Urological Pathology) Grade group of 4. The MED-diet with HIIT completion rate was 75% (9/12), whilst 90% of participants completed usual care (10/11). Figure 2 shows two participants allocated to the MED-diet with HIIT were deemed ineligible for HIIT and only completed weeks 0–12 of the MED-diet intervention due to high cardiac risk (*n* = 1), and already performing HIIT (*n* = 1). Attendance at the nutrition consults and exercise sessions was 100%. On five occasions, only two HIIT sessions were completed within one week (instead of the allocated three HIIT sessions), with participants completing four HIIT sessions the following week. No adverse events occurred from the MED-diet and HIIT interventions or outcome measures across the 20 weeks.

### 3.1. Intervention Adherence

MEDAS was superior in the MED-diet and HIIT group at 12 weeks [+4.9, (3.8, 5.9); *p* < 0.001] and 20 weeks [+2.8 (1.27, 4.45); *p* = 0.001) relative to the usual care group. The MED-diet and HIIT group showed high adherence to the nutrition prescription with 81% (*n* = 7/11) reaching ≥75% on the MEDAS at 12 weeks, and 66% (*n* = 6/9) at 20 weeks. The MED-diet and HIIT group showed significant reductions in energy intake [−1.7 MJ/day (−3.1, −0.2); *p* = 0.019], saturated fat [−14.2 g/day (−22.7, −5.6); *p* = 0.001], red meat [−0.5 servings/day (−0.9, −0.1); *p* = 0.016] and processed meat [−0.2 serves/day (−0.4, −0.1); *p* = 0.007], relative to the usual care group at 20 weeks (Table 2). The MED-diet and HIIT group significantly increased fibre [+7.8 g/day (1.9, 13.7); *p* = 0.010] and nuts and seeds [1.1 servings/day (0.2, 2.1); *p* = 0.013] relative to the usual care group at 20 weeks.

Adherence to the prescribed 85–95% HR_peak_ (based on the HR_peak_ achieved during the 12-week $\overset{\cdot }{V}$O_2peak_ test) was 93.4% for all four intervals over the 8-week training phase. Total time spent exercising at 85–95%% HR_peak_ was 86.5 ± 13.0% (13.8 ± 2.0 min) of the prescribed 16 min (4 × 4 min) across the 8 weeks. Average RPE was 15.6 ± 1.2 (equating to ~ “Hard”). HIIT performance outcomes revealed an average power output (80.4 ± 20.1 Watts), peak power output (225.4 ± 78.8 Watts), average revolutions per minute (RPM, 66.3 ± 7.3), peak RPM (87.3 ± 10.5), distance (16.0 ± 1.4 km) and estimated energy obtained from Wattbike (236.6 ± 67.2 kcal).

### 3.2. Cardiorespiratory Fitness

Table 3 shows there were no significant between-group differences in $\overset{\cdot }{V}$O_2peak_ (absolute and relative) at 12 weeks when the MED-diet and HIIT group was compared to usual care. However, after 20 weeks, the MED-diet and HIIT group showed a significant increase in absolute $\overset{\cdot }{V}$O_2peak_ [+0.3 L/min (0.1, 0.5); *p* = 0.002] and relative $\overset{\cdot }{V}$O_2peak_ [+4.9 mL·kg^−1^·min (2.5, 7.4); *p* < 0.001], compared to the usual care group.

### 3.3. Body Composition

Total body mass was significantly reduced at 12 weeks [−2.97 kg (−4.71, −1.24); *p* = 0.001] and 20 weeks [−3.32 kg (−5.10, −1.54); *p* < 0.001] when the MED-diet and HIIT group was compared to usual care. MED-diet and HIIT reduced lean muscle mass at 12 weeks [−1.35 kg (−2.75, 0.55); *p* = 0.060) and 20 weeks [−1.22 kg (−2.69, 0.25); *p* = 0.102). Fat mass was reduced at 12 weeks [−1.57 kg (−3.42, 0.28); *p* = 0.096] and 20 weeks [−1.25 kg (−3.14, 0.64); *p* = 0.192) from the MED-diet and HIIT relative to the usual care group.

### 3.4. Quality of Life

Quality of life as measured by the FACIT-G was significantly higher at 12 weeks when the MED-diet and HIIT group was compared to usual care [+9.2 points (2.7–15.8); *p* = 0.006]. After 20 weeks there was no significant differences between-groups in FACIT-G when the MED-diet and HIIT group was compared to usual care [+4.8 points (−2.0, 11.6); *p* = 0.167], yet these changes were clinically significant (i.e., ≥3 points). There were no further between-group differences in FACIT-G quality of life domains when the MED-diet and HIIT group was compared to usual care.

Quality of life, as measured by the SF-36, showed no significant between-group differences in the general health domain when the MED-diet and HIIT group was compared to usual care at 12 weeks [+4.1 points (−10.9, 19.1); *p* = 0.588] and 20 weeks [−3.1 points (−18.7, 12.4); *p* = 0.688]. Both the vitality [+16.2 points (6.2, 26.2); *p* = 0.002] and mental health composite domains [+4.1 points (0.1, 8.0); *p* = 0.042] showed significant improvements in favour of the MED-diet and HIIT group after 20 weeks compared to the usual care group.

## 4. Discussion

The present study has found that for men with prostate cancer undergoing ADT, a MED-diet with HIIT (i) significantly improves cardiorespiratory fitness relative to usual care at 20 weeks; (ii) reduces body weight relative to the usual care group at 20 weeks; (iii) maintains lean body mass despite achieving weight loss at 20 weeks; (iv) significantly improves vitality and mental health composite (SF-36) at 20 weeks, and clinical improvements were seen in prostate-cancer specific quality of life (FACIT-G) and cancer-related fatigue (FACIT-F) at 20 weeks relative to usual care; (v) is safe and feasible in men treated with ADT.

The MED-diet with HIIT showed a significant increase in $\overset{\cdot }{V}$O_2peak_ compared to the control group at 20 weeks (+4.9 mL·kg^−1^·min^−1^. *p* < 0.001; +0.3 L/min, *p* = 0.002). Given a 3.5 mL·kg^−1^·min^−1^ increase in cardiorespiratory fitness is associated with a 10% and 25% reduction in cancer specific mortality and cardiovascular-related mortality respectively [34], the increase of 4.9 mL·kg^−1^·min^−1.^ in $\overset{\cdot }{V}$O_2peak_ observed in our study potentially offers substantial cardiovascular health benefits. Our findings are consistent with a previous meta-analysis showing that HIIT is safe, feasible and efficacious in improving $\overset{\cdot }{V}$O_2peak_ (MD 3.73 mL·kg^−1^·min^−1^; *p* < 0.001) in adults after cancer treatment [11]. In men with prostate cancer, aerobic exercise interventions have shown moderate improvements in $\overset{\cdot }{V}$O_2peak_ (SMD 0.27 mL·kg^−1^·min^−1.^) [35], and recently, 12 weeks of HIIT (5–8 × 2 min 85–95% $\overset{\cdot }{V}$O_2peak_ treadmill speed and grade, with 2-min active recovery) during active surveillance has shown to significantly improve $\overset{\cdot }{V}$O_2peak_ compared to usual care (1.6 mL·kg^−1^·min^−1^; 95% CI, 0.3–2.9; *p* = 0.01) [36]. The substantial improvement in $\overset{\cdot }{V}$O_2peak_ in our study is likely explained by the high adherence to HIIT (86.5% of each interval was ≥85%HR_peak_), time spent exercise above ≥85%HR_peak_ (13.8 ± 2.0 min), and the low baseline $\overset{\cdot }{V}$O_2peak_. HIIT (4 × 4 min 85–95% HR_peak_) is therefore effective in improving cardiorespiratory fitness to a magnitude that is clinically meaningful in men treated with ADT, however further investigations in a larger sample, that is adequately powered, are required.

Combined nutrition and exercise interventions in men with prostate cancer have shown varied effects on reducing body weight; with weight loss ranging from 0.8kg to 6.1 kg [37]. The MED-diet significantly reduced weight (2.97 kg) and fat mass (1.57 kg); however, it also reduced lean mass (1.35 kg) after 12 weeks in this cohort of men treated with ADT [18]. The addition of HIIT to the MED-diet appears to preserve lean mass (−1.35 vs. −1.22 kg) and maintain total body mass (−2.97 vs. −3.32 kg) and fat mass (−1.57 vs. −1.25 kg) at 20 weeks. A recent systematic review in adults who have predominately finished cancer treatment suggests HIIT compared to moderate-intensity continuous training reduces body weight, but has no effect on lean mass [11]. Though two interventions (7 × 30 s > 85% HR_max_ [38] and 4 × 4 min 80–95% HR_max_ with 3 × 3 min active recovery [39]) have reported significant reductions in fat mass (ranging from 4 to 5.5%) following HIIT. In our study, it is likely that the significant reductions in body weight and fat mass from the MED-diet at 12 weeks, prior to the introduction of HIIT, diminished the potential changes in body composition from HIIT. Furthermore, our participants showed high adherence to the MED-diet and had similar energy intake across the 20 weeks. The MED-diet was hypocaloric, compared to habitual intake at baseline (−1.7 MJ at 12 weeks), with the MED-diet designed to meet individual estimated energy requirements. Herein, the fortnightly nutrition consultations progressive changed dietary intake to meet MED-diet targets and likely explains the substantial changes in body mass and composition occurring within the first 12 weeks and plateauing at 20 weeks when the participants are familiar with the MED-diet.

The MED-diet with HIIT showed inconsistent findings in cancer-related fatigue (SF−36 Vitality: +16.2 points, *p* = 0.001; FACIT-F: +2.6 points, *p* = 0.312) and quality of life (SF-36: −3.1 points; FACIT-G +8.2 points) compared to usual care at 20 weeks. However, relative to baseline, clinical improvements in the present study were observed in cancer-related fatigue and quality of life (≥3 points in the FACIT-F and G scale) from the MED-diet with HIIT after 20 weeks, and this suggests the combined diet and exercise prescription may provide quality of life benefits. Our findings contrast previous systematic reviews assessing HIIT interventions in cancer survivors [40], and combined exercise with or without nutrition interventions in men with prostate cancer [41]. These reviews have concluded that quality of life and cancer-related fatigue improve in response to exercise-only and exercise-plus-nutrition interventions. The usual care group in our study also improved in quality of life (+5.2 points) and cancer-related fatigue (+3.1 points) between 12 and 20 weeks. Whilst the reason for these improvements is not clear, men in the usual care group were free to seek exercise and/or dietary advice from other health care professionals, which may have influenced these outcomes. Given exercise interventions in prostate cancer have shown to improve quality of life and reduce cancer-related fatigue, further investigations in fatigued men with prostate cancer treated with ADT are warranted.

There are several strengths and limitations to our study. This is the first intervention to combine the MED-diet with HIIT and examine the synergistic effects on cardiorespiratory fitness, body composition and quality of life; all of which are known to be negatively impacted by ADT. This pilot study was highly supervised (fortnightly dietary interventions with 3 × 8 week supervised HIIT exercise) to ensure maximum safety and adherence across the 20-week intervention. However, there are some limitations worthy of comment. Our study failed to reach its sample size and experienced unique recruitment barriers which have been published elsewhere [18]. In addition, men in the intervention group had been diagnosed with prostate cancer longer than the control group (77.1 vs. 51.3 months) which may have influenced motivations and/or adherence to the intervention and the associated health benefits from face-to-face nutrition support and supervised exercise training. Although there were positive effects on body composition and quality of life measures from the MED-diet with HIIT relative to baseline, these results are masked by the efficacy of the 12-week MED-diet prior to starting HIIT. Thus, there was a ceiling effect on quality of life measures whereby the addition of HIIT to the MED-diet showed diminishing returns. Furthermore, it is unknown whether the MED-diet added any benefits when combined with HIIT for cardiorespiratory fitness. To test whether there are additive benefits from the MED-diet on HIIT, factorial 2 × 2 design RCT with four groups (MED-diet vs. HIIT vs. MED-diet with HIIT vs. usual care) would be required in future studies. Lastly, whether the improvements in cardiorespiratory fitness are associated with improvements in cardiovascular health (i.e., cholesterol, triglycerides) from the MED-diet with HIIT warrants investigation.

## 5. Conclusions

In summary, the HIIT component of our intervention showed significant improvements in cardiorespiratory fitness to a magnitude that is associated with reduced cardiovascular-related morbidity and mortality in adults with cancer. Whilst the MED-diet likely offered cardiovascular benefits with HIIT, the physiological mechanism/s is/are unknown, and should be considered in future larger-scale investigations to extend our findings. The MED-diet with HIIT showed significant reductions in body weight, which is typically increased by ADT, and may be of clinical benefit for future weight loss interventions. Cancer-related fatigue and quality of life was improved after 12 weeks of the MED-diet; however, there were diminishing returns when measured at 20 weeks, after HIIT had been introduced. Future larger-scale trials examining the MED-diet with HIIT on cardiorespiratory fitness, body composition and quality of life are warranted to extend our findings.

## Figures and Tables

**Table 1:** Baseline participant characteristics.

	All (*n* = 23)	Intervention (*n* = 12)	Usual Care (*n* = 11)
Age (years)	65.9 ± 7.8	66.6 ± 7.6	65.1 ± 7.9
Height (cm)	177.7 ± 6.7	177.2 ± 7.5	178.3 ± 5.7
BMI (kg/m^2^)	28.9 ± 3.4	27.4 ± 3.0	30.6 ± 2.9
Married (%)	56.5	54.5	58.3
Past smoker (%)	56.5	63.3	58.3
Meeting PA guidelines (%)	43.4	27.2	58.3
Gleason Score	8.4 ± 1.1	8.1 ± 0.9	8.7 ± 1.2
Prostate Specific Antigen concentration (ng/mL)	1.1 ± 1.3	1.3 ± 1.2	1.0 ± 1.3
Time since diagnosis (months)	64.8 ± 53.2	77.1 ± 58.8	51.3 ± 42.4
Time on ADT (months)	33.8 ± 35.6	36.4 ± 38.3	31.0 ± 32.2
Previous radiation (%)	65.2	66.6	63.6
Duration of radiation (months)	2.2 ± 1.6	2.4 ± 2.1	2.0 ± 0.1
Previous chemotherapy (%)	17.3	16.6	18.1
Duration of chemotherapy (months)	3.2 ± 1.6	4.0 ± 2.0	2.5 ± 0.5
Previous prostatectomy (%)	30.4	8.6	45.4

Data are presented as mean ± SD or %. ADT: androgen deprivation therapy; BMI: body mass index; PA: physical activity, as measured by the Godin Leisure-Time Physical Activity Questionnaire [33].

**Table 2:** The effects of the MED-diet and HIIT compared to usual care on energy, macronutrients and foods groups.

	Group	*n*	Baseline	*p* Value	*n*	12 Weeks	*p* Value (0 vs. 12 Weeks)	*p* Value ^a^ (INT vs. UC)	*n*	20 Weeks	*p* Value (0 vs. 20 Weeks)	*p* Value (12 vs. 20 Weeks)	*p* Value ^b^ (INT vs. UC)
Energy (MJ/day)	INT	12	10.3 (9.1, 10.9)	0.689	11	8.6 (7.6, 9.5)	0.032	0.057	9	8.2 (7.2, 9.2)	0.014	>0.999	0.019
	UC	11	9.7 (8.8, 10.7)		10	9.9 (8.9, 10.9)	>0.999		10	9.9 (8.9, 10.9)	>0.999	>0.999	
Protein (g/day)	INT	12	110.1 (99.7, 120)	0.601	11	108.8 (97.9, 119)	>0.999	0.850	9	103.7 (91.7, 115.7)	>0.999	>0.999	0.211
	UC	11	106.1 (95.2, 117)		10	107.3 (95.9, 118)	>0.999		10	114.2 (102.8, 125.6)	>0.999	>0.999	
Fat (g/day)	INT	12	91.7 (78.7, 104)	0.776	11	76.5 (63.0, 90.0)	0.316	0.048	9	79.8 (65.1, 94.5)	0.766	>0.999	0.190
	UC	11	89.0 (75.4, 102)		10	96.3 (82.1, 110)	>0.999		10	93.4 (79.2, 107.5)	>0.999	>0.999	
Saturated fat (g/day)	INT	12	33.6 (28.7, 38.9)	0.477	11	17.2 (11.6, 22.8)	<0.001	<0.001	9	20.6 (14.5, 26.7)	0.002	0.674	0.001
	UC	11	30.8 (24.8, 36.4)		10	34.0 (28.1, 39.9)	>0.999		10	34.8 (28.9, 40.7)	>0.999	>0.999	
Polyunsaturated fat (g/day)	INT	12	15.9 (12.5, 19.2)	0.952	11	20.8 (17.4, 24.3)	0.043	0.065	9	18.4 (14.8, 22.1)	0.183	0.222	0.280
	UC	11	16.0 (12.5, 19.5)		10	16.2 (12.6, 19.8)	>0.999		10	15.6 (11.9, 19.3)	0.833	0.765	
Monounsaturated fat (g/day)	INT	12	39.2 (31.5, 47.0)	0.770	11	37.5 (29.5, 45.5)	>0.999	0.442	9	36.0 (27.2, 44.7)	0.526	0.767	0.763
	UC	11	40.9 (32.9, 48.9)		10	42.0 (33.6, 50.4)	>0.999		10	34.1 (25.3, 42.9)	0.198	0.140	
LCN3FA (g/day)	INT	12	0.80 (0.43, 1.18)	0.800	11	1.42 (1.03, 1.81)	0.033	0.013	9	1.2 (0.7, 1.6)	0.090	0.344	0.145
	UC	11	0.73 (0.34, 1.13)		10	0.69 (0.28, 1.10)	>0.999		10	0.7 (0.3, 1.1)	0.943	0.803	
ALA (g/day)	INT	12	1.71 (0.80, 2.61)	0.984	11	2.68 (1.74, 3.62)	0.272	0.184	9	3.1 (2.1, 4.1)	0.013	0.483	0.587
	UC	11	1.72 (0.78, 2.66)		10	1.77 (0.79, 2.75)	>0.999		10	1.7 (0.7, 2.7)	0.933	0.998	
Linoleic acid (g/day)	INT	12	13.0 (10.4, 15.6)	0.917	11	15.4 (12.7, 18.0)	0.573	0.301	9	14.1 (11.2, 16.9)	0.479	0.392	0.490
	UC	11	13.2 (10.5, 15.9)		10	13.3 (10.6, 16.1)	>0.999		10	12.6 (9.7, 15.5)	0.725	0.648	
Cholesterol (g/day)	INT	12	338 (259, 417)	0.369	11	316 (233, 399)	>0.999	0.834	9	244 (153, 335)	0.104	0.221	0.244
	UC	11	286 (203, 368)		10	303 (217, 390)	>0.999		10	320 (229, 412)	0.554	0.774	
Carbohydrates (g/day)	INT	12	238 (209, 267)	0.820	11	197 (167, 227)	0.064	0.231	9	180.0 (147.3, 212.6)	0.009	0.978	0.065
	UC	11	233 (203, 264)		10	224 (192, 256)	>0.999		10	222.9 (191.2, 254.6)	>0.999	>0.999	
Fibre (g/day)	INT	12	34.6 (30.8, 38.4)	0.976	11	47.0 (43.0, 50.9)	<0.001	<0.001	9	44.7 (40.5, 49.0)	<0.001	0.330	0.010
	UC	11	34.6 (30.6–38.5)		10	34.9 (30.8, 39.0)	>0.999		10	36.9 (32.7, 41.0)	>0.999	>0.999	
Ethanol (g/day)	INT	12	15.3 (9.26, 21.5)	0.401	11	4.9 (1.4, 11.3)	0.018	0.051	9	3.2 (1.3, 10.0)	0.002	0.635	0.012
	UC	11	13.4 (6.96, 19.8)		10	14.2 (7.51, 20.9)	>0.999		10	15.6 (8.9, 22.3)	0.551	0.706	
Fruit (servings/day)	INT	12	2.21 (1.68, 2.75)	0.960	11	2.46 (1.91, 3.01)	>0.999	0.019	9	2.3 (1.7, 2.9)	>0.999	>0.999	0.223
	UC	11	2.23 (1.68, 2.79)		10	1.50 (0.92, 2.07)	0.122		10	1.7 (1.2, 2.3)	0.640	0.986	
Vegetables (servings/day)	INT	12	4.28 (3.33, 5.22)	0.943	11	8.22 (7.24, 9.20)	<0.001	0.001	9	7.3 (6.3, 8.2)	<0.001	0.328	0.061
	UC	11	4.23 (3.42, 5.22)		10	4.84 (3.81, 5.87)	0.872		10	5.9 (4.9, 7.0)	0.020	0.223	
Refined grain (servings/day)	INT	12	3.35 (2.40, 4.30)	0.690	11	1.92 (0.93, 2.90)	0.090	0.011	9	2.0 (0.9, 3.1)	0.134	>0.999	0.487
	UC	11	3.62 (2.64, 4.61)		10	3.81 (2.77, 4.85)	>0.999		10	2.5 (1.5, 3.5)	0.391	>0.999	
Nuts and seeds (servings/day)	INT	12	0.92 (0.33, 1.51)	0.401	11	1.89 (1.28, 2.51)	0.114	0.717	9	1.7 (1.0, 2.3)	0.376	>0.999	0.013
	UC	11	1.28 (0.67, 1.90)		10	1.73 (1.08, 2.37)	0.893		10	0.5 (0.1, 1.1)	0.336	0.036	
Fish (servings/day)	INT	12	0.61 (0.38, 0.84)	0.897	11	0.90 (0.67, 1.13)	0.121	0.006	9	0.7 (0.4, 0.9)	0.904	0.295	0.702
	UC	11	0.59 (0.35, 0.83)		10	0.44 (0.20, 0.67)	>0.999		10	0.6 (0.4, 0.8)	>0.999	>0.999	
Red meat (servings/day)	INT	12	0.75 (0.48, 1.03)	0.398	11	0.33 (0.06, 0.61)	0.071	0.205	9	0.2 (0.0, 0.5)	0.038	>0.999	0.016
	UC	11	0.59 (0.31, 0.86)		10	0.60 (0.30, 0.89)	>0.999		10	0.8 (0.4, 1.1)	>0.999	>0.999	
Processed meat (servings/day)	INT	12	0.36 (0.25, 0.47)	0.632	11	0.04 (0.01, 0.15)	<0.001	<0.001	9	0.0 (0.0, 0.2)	>0.999	0.001	0.007
	UC	11	0.32 (0.21, 0.43)		10	0.38 (0.27, 0.49)	>0.999		10	0.3 (0.2, 0.4)	>0.999	>0.999	
Poultry (servings/day)	INT	12	0.43 (0.20, 0.65)	0.985	11	0.47 (0.25, 0.70)	>0.999	0.669	9	0.6 (0.3, 0.8)	>0.999	>0.999	0.078
	UC	11	0.42 (0.20, 0.65)		10	0.40 (0.16, 0.64)	>0.999		10	0.2 (0.0, 0.5)	>0.999	>0.999	
Dairy (servings/day)	INT	12	2.84 (2.12, 3.56)	0.353	11	1.58 (0.83, 2.33)	0.033	0.508	9	1.7 (0.9, 2.6)	0.136	>0.999	0.248
	UC	11	2.35 (1.60, 3.10)		10	1.95 (1.16, 2.74)	>0.999		10	2.4 (1.6, 3.2)	>0.999	>0.999	

Intention to treat linear mixed modelling analysis; fixed factors: group, time, group × time; fixed covariates: baseline variable score; random factors: participants. Abbreviations: ALA, α-linoleic acid; LCN3FA, long chain ω-3 fatty acid. INT, Intervention; UC, Usual care. All values are mean (95%CI). ^a^ Between group comparisons at 12 weeks. ^b^ Between group comparisons at 20 weeks.

**Table 3:** The effects of the MED-diet and HIIT compared to usual care on cardiorespiratory fitness, body composition and quality of life.

	Group	*n*	Baseline	*p* Value	*n*	12 Weeks	*p* Value (0 vs. 12 Weeks)	*p* Value ^a^ (INT vs. UC)	*n*	20 Weeks	*p* Value (0 vs. 20 Weeks)	*p* Value (12 vs. 20 Weeks)	*p* Value ^b^ (INT vs. UC)
**Cardiorespiratory fitness**													
VO_2peak_ (L/min)	INT	12	2.02 (1.89, 2.16)	0.740	11	2.07 (1.94, 2.21)	0.521	0.492	9	2.34 (2.19, 2.48)	<0.001	<0.001	0.002
	UC	11	2.06 (1.93, 2.19)		10	2.00 (1.87, 2.14)	0.487		10	2.02 (1.88, 2.15)	0.608	0.857	
VO_2peak_ (mL·kg^−1.^min^−1^)	INT	12	22.1 (20.4, 23.7)	0.961	11	23.6 (21.9, 25.2)	0.104	0.161	9	26.8 (25.0, 28.5)	<0.001	<0.001	<0.001
	UC	11	22.0 (20.5, 23.9)		10	21.9 (20.2, 23.5)	0.874		10	21.8 (20.1, 23.5)	0.821	0.947	
400 m walk test (s)	INT	12	260.6 (241.3, 279.8)	0.872	11	246.0 (226.2, 265.8)	0.103	0.945	9	241.0 (220.6, 261.4)	0.203	0.589	0.457
	UC	11	268.6 (248.4, 288.8)		10	247.0 (226.8, 267.2)	0.106		10	251.9 (231.7, 272.1)	0.064	0.585	
**Body composition (kg)**													
Total body mass	INT	12	92.0 (90.9,93.1)	0.784	11	88.7 (87.5, 89.8)	<0.001	0.001	9	87.8 (86.5, 89.0)	<0.001	0.371	<0.001
	UC	11	92.2 (91.1, 93.4)		10	91.6 (90.4, 92.8)	>0.999		10	91.1 (89.9, 92.3)	0.461	>0.999	
Lean muscle mass	INT	12	53.2 (52.2, 54.1)	0.750	11	52.0 (51.0, 53.0)	0.397	0.060	9	52.0 (50.9, 53.0)	0.397	>0.999	0.102
	UC	11	53.4 (52.4, 54.3)		10	53.4 (52.3, 54.4)	>0.999		10	53.2 (52.2, 54.2)	>0.999	>0.999	
Fat mass	INT	12	29.5 (28.3, 30.7)	0.696	11	27.8 (26.6, 29.0)	0.032	0.096	9	27.2 (25.9, 28.5)	0.005	0.796	0.192
	UC	11	29.8 (28.6, 31.1)		10	29.3 (28.1, 30.6)	>0.999		10	28.5 (27.2, 29.7)	0.206	0.178	
**FACIT ^1^**													
General (0–108)	INT	12	83.1 (78.7, 87.4)	0.888	11	90.5 (85.9, 95.0)	0.038	0.006	9	91.3 (86.4, 96.3)	0.032	>0.999	0.167
	UC	11	82.6 (78.1, 87.2)		10	81.2 (76.5, 86.0)	>0.999		10	86.5 (81.8, 91.3)	0.569	0.343	
Total (0–160)	INT	12	120.3 (113.8, 26.9)	0.771	11	133.4 (126.6, 140.3)	0.013	0.002	9	133.8 (126.3, 141.3)	0.015	>0.999	0.141
	UC	11	118.9 (112.1, 125.8)		10	117.1 (110.0, 124.3)	>0.999		10	126.1 (118.9, 133.2)	0.515	0.270	
TOI (0–108)	INT	12	83.2 (77.1, 89.2)	0.762	11	91.9 (85.6, 98.2)	0.081	0.024	9	91.5 (84.7, 98.4)	0.132	>0.999	0.371
	UC	11	81.8 (75.5, 88.2)		10	81.1 (74.5, 87.8)	>0.999		10	87.1 (80.5, 93.8)	0.739	0.633	
Fatigue (0–52)	INT	12	37.1 (33.9, 40.3)	0.684	11	42.9 (39.6, 46.3)	0.025	0.005	9	42.5 (38.8, 46.1)	0.064	>0.999	0.312
	UC	11	36.2 (32.8, 39.5)		10	35.7 (32.2, 39.3)	>0.999		10	39.8 (36.3, 43.4)	0.378	0.317	
Physical Wellbeing (0–28)	INT	12	23.3 (21.9, 24.7)	0.614	11	25.1 (23.6, 26.6)	0.145	0.036	9	25.0 (23.4, 26.6)	0.206	>0.999	0.247
	UC	11	22.8 (21.3, 24.6)		10	22.8 (21.3, 24.4)	>0.999		10	23.7 (22.2, 25.3)	>0.999	>0.999	
Social Wellbeing (0–28)	INT	12	19.7 (17.4, 22.1)	0.889	11	21.9 (19.5, 24.4)	0.666	0.052	9	23.1 (20.4, 25.7)	0.214	>0.999	0.078
	UC	11	20.0 (17.5, 22.4)		10	18.4 (15.9, 21.0)	>0.999		10	19.7 (17.2, 22.3)	>0.999	>0.999	
Emotional Wellbeing (0–24)	INT	12	18.5 (17.3, 19.7)	0.964	11	20.8 (19.5, 22.0)	0.024	0.083	9	20.5 (18.6, 21.4)	0.256	0.798	0.320
	UC	11	18.5 (17.2, 19.7)		10	19.1 (17.8, 20.5)	0.804		10	21.0 (19.7, 22.4)	0.015	0.125	
Functional Wellbeing (0–28)	INT	12	21.5 (19.8, 23.3)	0.747	11	22.5 (20.7, 24.4)	>0.999	0.177	9	23.2 (21.1, 25.2)	0.894	>0.999	0.295
	UC	11	21.1 (19.3, 23.0)		10	20.7 (18.7, 22.6)	>0.999		10	21.7 (19.7, 23.6)	>0.999	>0.999	
**SF-36 ^1^**													
Physical Function (0–100)	INT	12	80.8 (71.7, 89.9)	0.617	11	78.3 (68.8, 87.7)	0.680	0.571	9	89.7 (79.3, 100.1)	0.669	0.498	0.304
	UC	11	77.4 (67.9, 87.0)		10	82.2 (72.2, 92.1)	>0.999		10	82.2 (72.2, 92.1)	>0.999	>0.999	
Role Function (0–100)	INT	12	72.6 (64.8, 80.4)	0.438	11	72.8 (64.7, 80.9)	>0.999	0.685	9	80.7 (71.8, 89.6)	0.616	0.616	0.858
	UC	11	68.1 (60.0, 76.3)		10	75.2 (66.7, 83.8)	0.510		10	79.6 (71.1, 88.2)	0.141	0.811	
Bodily Pain (0–100)	INT	12	76.5 (65.6, 87.3)	0.622	11	77.8 (66.3, 89.1)	>0.999	0.895	9	80.3 (68.1, 92.5)	>0.999	>0.999	0.407
	UC	11	72.5 (61.2, 83.9)		10	76.8 (65.0, 88.6)	>0.999		10	73.2 (61.4, 85.0)	>0.999	>0.999	
General (0–100)	INT	12	57.2 (47.2, 67.3)	0.737	11	66.3 (55.9, 76.7)	0.710	0.588	9	59.7 (48.5, 70.9)	>0.999	>0.999	0.688
	UC	11	59.7 (49.2, 70.2)		10	62.2 (51.3, 73.1)	>0.999		10	62.9 (52.0, 73.8)	>0.999	>0.999	
Vitality (0–100)	INT	12	58.7 (52.4, 65.0)	0.966	11	71.6 (64.5, 77.7)	0.001	0.003	9	75.9 (68.7, 83.1)	0.015	0.838	0.002
	UC	11	58.5 (51.9, 65.1)		10	56.5 (49.6, 63.4)	0.645		10	59.7 (52.8, 66.6)	>0.999	>0.999	
Social Functioning (0–100)	INT	12	86.1 (79.0, 93.1)	0.733	11	87.1 (79.8, 94.4)	>0.999	0.138	9	91.8 (83.9, 99.6)	>0.999	>0.999	0.482
	UC	11	84.3 (77.0, 91.7)		10	79.1 (71.4, 86.7)	0.820		10	87.8 (80.2, 95.5)	>0.999	0.230	
Role Emotion (0–100)	INT	12	79.8 (73.4, 86.2)	0.893	11	85.4 (78.7, 92.1)	0.521	0.780	9	94.4 (87.0, 101.9)	0.024	0.385	0.230
	UC	11	79.2 (72.5, 85.9)		10	84.1 (77.0, 91.1)	>0.999		10	88.2 (81.2, 95.3)	0.407	>0.999	
Mental Health (0–100)	INT	12	80.3 (75.7, 84.8)	0.925	11	84.4 (79.6, 89.2)	0.748	0.071	9	88.1 (82.9, 93.4)	0.112	0.785	0.127
	UC	11	80.0 (75.2, 84.7)		10	78.0 (73.0, 83.0)	>0.999		10	82.5 (77.5, 87.5)	>0.999	>0.999	
PH Composite (0–100)	INT	12	48.6 (45.1, 52.0)	0.649	11	48.7 (45.1, 52.3)	>0.999	0.737	9	50.0 (46.1, 54.0)	>0.999	>0.999	0.695
	UC	11	47.4 (43.8, 51.0)		10	49.6 (45.8, 53.4)	>0.999		10	49.0 (45.2, 52.8)	>0.999	>0.999	
MH Composite (0–100)	INT	12	51.6 (49.1, 54.1)	0.923	11	55.2 (52.6, 57.8)	0.188	0.011	9	57.6 (54.7, 60.4)	0.015	0.704	0.042
	UC	11	51.6 (49.0, 54.2)		10	50.3 (47.6, 53.0)	>0.999		10	53.4 (50.7, 56.2)	>0.999	0.656	

Intention to treat linear mixed modelling analysis; fixed factors: group, time, group × time; covariates: baseline variable score; random factors: participants. Abbreviations: FACIT, functional assessment of chronic illness therapy; INT, Intervention; MH, Mental health; PH, Physical health; SF-36, The Medical Outcomes Study 36-Item Short-Form Health Survey; TOI, trial outcome index (sum of physical, functional, and ‘additional concerns’ subscales); UC = usual care. All values are mean (95%CI). ^1^ Note higher scores = higher quality of life; for symptom scales (i.e., fatigue), higher scores = less symptoms. ^a^ Between group comparisons at 12 weeks. ^b^ Between group comparisons at 20 weeks.

## Data Availability

Data can be made available upon request.
